# Quantum machine learning

**DOI:** 10.1093/nsr/nwy149

**Published:** 2018-11-30

**Authors:** Jonathan Allcock, Shengyu Zhang

**Affiliations:** Tencent Quantum Laboratory, China

## INTRODUCTION

Quantum machine learning is at the crossroads of two of the most exciting current areas of research: quantum computing and classical machine learning. Although the field is still in its infancy, the body of literature is already large enough to warrant several review articles [1–3]. This short survey focuses on a selection of significant recent results on the subtopic of quantum neural networks, an area that hopes to build on the enormous impact that classical neural networks have had. In particular, we concentrate on quantum generalizations of popular neural-network concepts such as Boltzmann machines, generative adversarial networks and autoencoders, as well as applications of classical neural networks to quantum information and vice versa.

## BOLTZMANN MACHINE NEURAL NETWORKS

**Figure 1.**
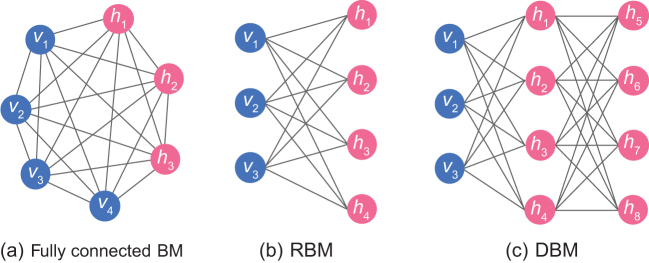
Examples of Boltzmann machines. In a fully connected Boltzmann machine, connections exist between all visible and hidden neurons. In a restricted Boltzmann machine (RBM), there are no connections between neurons of the same type. A deep Boltzmann machine (DBM) consists of adding one or more additional layers of hidden neurons to an RBM, with no connections between neurons in the same layer.

The neural networks that have received most recent attention from a quantum perspective are known as Boltzmann machines (BMs). BMs consist of binary neurons divided into visible and hidden units, represented by vectors $v$ and *h* respectively (see Fig. 1). To every assignment of the visible and hidden neurons, a BM associates a joint probability

\begin{equation*}
P(v,h) = \frac{1}{Z}e^{-E(v,h)},
\end{equation*}

where *Z* is a normalization factor, *E*($v$, *h*) is a quadratic energy function of $v$ and *h*, and there is a set $\mathcal {W}$ of real-valued coefficients in the energy function that are weight parameters to be learned. The training process consists of using methods such as gradient descent to optimize the weights so as to maximize the likelihood that *P*($v$) = ∑_*h*_*P*($v$, *h*) reproduces the statistics of observed data.

### Quantum Boltzmann machines

The energy-based nature of BMs gives a natural framework for considering quantum generalizations of their behavior. The BM energy function is equivalent to the Hamiltonian of a simple Ising model and one might hope that more general Hamiltonians allowed by quantum mechanics could explain certain data sets better than classically.

Amin *et al.* [4] adopt this approach, and take the so-called transverse field Ising Hamiltonian as the energy function. This increased generality brings certain difficulties, however, as the transverse field term makes training the quantum Boltzmann machine (QBM) non-trivial. Although the general scaling and performance of QBMs is not yet well understood, for certain small-size examples the authors observe that their QBMs can learn data distributions better than classical BMs.

Kieferova and Wiebe [5] extend the formalism of Amin *et al.* and consider more general Hamiltonians that, unlike the transverse Ising model, are not in general believed to be classically efficiently simulatable. Furthermore, they propose new methods for training that allow for parameters corresponding to quantum terms to be learned. Finally, their QBM enables a form of quantum-state tomography that can learn non-classical data, and also generate copies of the reconstructed state. Again, small-scale numerical evidence suggests that their proposals may provide an advantage over classical BMs, although their tendency to overfit data must be investigated further.

### Quantum algorithms for training classical Boltzmann machines

The practical value of BMs, either classical or quantum, relies in part on the ease with which they are trained. This in turn depends on sampling efficiently from the Boltzmann distribution. Classically this can be done approximately only for the special case of restricted Boltzmann machines (RBM), i.e. where connections are between visible and hidden units, and no connections exist between neurons of the same type.

Wiebe *et al.* [6] propose two quantum algorithms for Boltzmann sampling, based on starting from a state corresponding to the product distribution that is closest to the target Boltzmann distribution. While small-scale numerical studies are encouraging, true bounds on the running time of their algorithms rely on a problem-dependent parameter κ, the scaling of which is hard to estimate. The hope remains though that empirically their algorithms work well in practice, even for multi-layer and fully connected BMs.

### Classical algorithms with quantum Boltzmann machine states

The expressive power of BMs for compactly representing complicated probability distributions has led to another line of investigation, namely their use for representing quantum many-body wave functions.

Carleo and Troyer [7] introduce this idea by expressing a quantum state of *N* spins as

\begin{equation*}
\vert \Psi \rangle = \sum _v \Psi (v,\mathcal {W})\vert v \rangle ,
\end{equation*}

where the coefficients $\Psi (v,\mathcal {W}) = \sum _h e^{-E(v,h)}$ are defined by an RBM with *N* visible units, *M* hidden units, and complex-valued network weights $\mathcal {W}$. Given a many-body system Hamiltonian *H*, the task is then to use classical machine learning to find the network parameters that either minimize the energy 〈Ψ|*H*|Ψ〉/〈Ψ|Ψ〉 or best reproduce unitary dynamics $iH\vert \Psi (t) \rangle = \frac{d}{dt}\vert \Psi (t) \rangle$.

The authors apply this technique to two prototypical spin models and achieve high accuracy in both ground-state estimation and dynamical evolution, improving on existing state-of-the-art techniques. Their results indicate that RBM states can capture complex many-body correlations in an efficient manner—evidence reinforced by the work of Deng *et al.* [8], who show that certain topological quantum states in one, two and three dimensions can be represented by RBM states with a number of parameters only linear in the system size.

In a subsequent study, however, Gao and Duan [9] prove that although RBM states can indeed represent many highly entangled states, their expressive power is limited in that they cannot represent arbitrary states generated by polynomial-sized quantum circuits, nor those that are the ground states of *k*-local Hamiltonians with polynomial-sized energy gaps, unless the polynomial hierarchy from computational complexity theory collapses. On the other hand, they show that these two important classes of states can be efficiently represented by a deep Boltzmann machine of three layers. Intriguingly though, another paper by Deng *et al.* [10] gives an analytic construction of a family of RBM states with a number of parameters linear in the system size that exhibits maximal volume-law entanglement, and that cannot be represented efficiently by either matrix product states (MPS) or tensor-network (TN) states. This shows that the expressive power of even efficiently parametrized RBM states is not limited by entanglement in the same way that other popular compact representations are. This gives the hope that RBM states may prove a valuable tool in understanding entangled quantum systems intractable by traditional methods.

## DEEP LEARNING AND ENTANGLEMENT

Levine *et al.* [11] establish another connection between the many-body wave function and neural networks, this time in the form of convolutional arithmetic circuits (ConvAC), a variant of the popular convolutional neural networks in widespread use today.

By proving a one-to-one equivalence between a ConvAC circuit and TN quantum states, the authors are able to use tools from quantum information theory to analyze the entanglement present in the corresponding TN, and quantify the neural network's ability to model complex correlations between its inputs. This in turn can be used to obtain bounds on the expressivity of a given ConvAC architecture and be of practical use in choosing network parameters tailored to the data. While ConvAC are not widely used in practice, the authors empirically validate that the insights gained can be applied successfully to more common convolutional neural-network designs.

## QUANTUM GENERATIVE ADVERSARIAL NETWORKS

Generative adversarial networks (GAN) are machine-learning algorithms in which two players—a generator and a discriminator—compete against each another. The generator's goal is to create and send fake data samples to the discriminator and maximize the probability that the discriminator erroneously believes that the sample comes from some true data distribution. The discriminator attempts to discern real from fake data as best as possible. Under reasonable assumptions the game converges to a point where the discriminator is unable to discriminate with probability greater than 1/2 (i.e. no better than random guessing), and the generator in fact has learned to generate data from the true distribution.

Lloyd and Weedbrook [12] consider the natural extension in which one or both of the generator and discriminator are equipped with quantum information processors. When the data to be learned are a quantum state and both parties have quantum processors, the authors show that the unique end point of the adversarial game also coincides with the generator producing a quantum state corresponding to the true data statistics, and the discriminator unable to discern true from false data better than random guessing. They further argue that a different outcome may hold when the true samples are produced by the measurement of some quantum state, and the generator is purely classical. The concept of quantum supremacy is based on the assumption that there exist probability distributions accessible to a quantum computer that no classical computer can efficiently sample from. If the true data correspond to one of these distributions, then no classical generator can efficiently generate fake data reproducing the true statistics and, in principle, the discriminator can successfully discriminate true from fake data. However, whether the discriminator can efficiently find the measurements required to do so is unclear.

## COMPRESSION VIA QUANTUM AUTOENCODERS

Quantum-computing techniques have also found applications in autoencoders—neural networks where a larger number of input and output neurons are connected via a smaller number of intermediary latent neurons. The goal is to tune the weights of the network so that a training set of classical bit strings can be approximately recovered after being passed through the autoencoder.

Romero *et al.* [13] consider instead a training set of quantum states, and ask whether a quantum circuit with a similar bottleneck structure can compress and approximately recover the data. The circuits that they consider consist of gates chosen from some set of parametrized unitary operations, and arranged in some fixed order. The parameters are then optimized classically so that the circuit best predicts the training-set labels. The authors demonstrate the validity of this approach by showing that ground states of the Hubbard model and certain molecules can indeed be compressed and recovered by such circuits. However, in general, the performance of such quantum autoencoders will be highly dependent on the kind of programmable circuit on which they are based, and at present there does not seem to be a structured way of determining this.

## OUTLOOK

Quantum neural networks are still in their infancy and, while the above works are important steps towards bridging our understanding between classical neural networks and quantum computing, many questions remain unanswered. If the expressivity of Boltzmann machine states is not restricted by entanglement in the same way that MPS and tensor-network states are, what is the best way of understanding their limitations? How can physicists use the correspondence between ConvAC and TN to their advantage in finding efficient, expressive representations of many-body wave functions? Can classical data distributions be learned more efficiently by quantum GAN than classical GAN? Whatever the outcome of these and many other related questions, this promises to be an exciting and active area of research in the years to come.
